# Demonstration of constant nitrogen and energy amounts in pig urine under acidic conditions at room temperature and determination of the minimum amount of hydrochloric acid required for nitrogen preservation in pig urine

**DOI:** 10.5713/ab.22.0243

**Published:** 2022-11-13

**Authors:** Jongkeon Kim, Bokyung Hong, Myung Ja Lee, Beob Gyun Kim

**Affiliations:** 1 Department of Animal Science and Technology, Konkuk University, Seoul 05029, Korea

**Keywords:** Hydrochloric Acid, Nitrogen Preservation, Pig Urine

## Abstract

**Objective:**

The objectives were to demonstrate that the nitrogen and energy in pig urine supplemented with hydrochloric acid (HCl) are not volatilized and to determine the minimum amount of HCl required for nitrogen preservation from pig urine.

**Methods:**

In Exp. 1, urine samples of 3.0 L each with 5 different nitrogen concentrations were divided into 2 groups: 1.5 L of urine added with i) 100 mL of distilled water or ii) 100 mL of 6 *N* HCl. The urine in open plastic containers was placed on a laboratory table at room temperature for 10 d. The weight, nitrogen concentration, and gross energy concentration of the urine samples were determined every 2 d. In Exp. 2, three urine samples with different nitrogen concentrations were added with different amounts of 6 *N* HCl to obtain varying pH values. All urine samples were placed on a laboratory table for 5 d followed by nitrogen analysis.

**Results:**

Nitrogen amounts in urine supplemented with distilled water decreased linearly with time, whereas those supplemented with 6 *N* HCl remained constant. Based on the linear broken-line analysis, nitrogen was not volatilized at a pH below 5.12 (standard error = 0.71 and p<0.01). In Exp. 3, an equation for determining the amount of 6 *N* HCl to preserve nitrogen in pig urine was developed: additional 6 *N* HCl (mL) to 100 mL of urine = 3.83×nitrogen in urine (g/100 mL)+0.71 with R^2^ = 0.96 and p<0.01. If 62.7 g/d of nitrogen is excreted, at least 240 mL of 6 *N* HCl should be added to the urine collection container.

**Conclusion:**

Nitrogen in pig urine is not volatilized at a pH below 5.12 at room temperature and the amount of 6 *N* HCl required for nitrogen preservation may be up to 240 mL per day for a 110-kg pig depending on urinary nitrogen excretion.

## INTRODUCTION

Metabolizable energy (ME) in feeds has been widely employed in swine diet formulations [1] as energy utilization is better reflected in the ME system compared with the gross energy (GE) or digestible energy (DE) system. Feed ME values are determined by subtracting urinary and gaseous energy from ingested DE. In this calculation, gaseous energy is often neglected due to the small quantity in pigs. Thus, an accurate measurement of urinary energy is essential for determining ME values in feeds [2–5].

Energy in the pig urine consists mainly of urea which can be hydrolyzed to ammonia and evaporated into the air [6]. As the prevention of ammonia volatilization from urine is essential for an accurate ME determination, the addition of acids in the urine collection containers is a general practice to keep the urine acidic [7,8]. However, the amounts of acids used for nitrogen preservation vary among experiments [4,9–11]. To our knowledge, little information is available on the amount of hydrochloric acid (HCl) required for nitrogen preservation in pig urine. Therefore, the objectives of the present experiments were to demonstrate that nitrogen and energy in pig urine are not volatilized under acidic conditions and to determine the amounts of HCl required for nitrogen preservation.

## MATERIALS AND METHODS

### Animal care

The experimental protocol was approved by the Institutional Animal Care and Use Committee at Konkuk University, Republic of Korea (KU17049 and KU19058).

### Exp. 1. Nitrogen and energy contents in pig urine under acidic condition

Urine samples were collected from 5 barrows (Landrace× Yorkshire) with a mean body weight (BW) of 68.1±4.0 kg for 24 h with no acid in the urine collection containers and were filtered using cotton cloth (0.5 mm pore size) to remove impurities. The samples were stored in a sealed container at −20°C. Nitrogen concentrations in the urine samples were 0.29%, 0.58%, 0.63%, 0.66%, and 0.68%. Each urine sample (approximately 3.0 L) was divided into 2 groups of 1.5 L which were supplemented with either 100 mL of 6 *N* HCl to obtain a pH below 2 or 100 mL of distilled water. Each 200 mL urine sample added with HCl, or distilled water was placed in a plastic container. All plastic containers with the urine samples were placed on a laboratory table for 10 d at room temperature of 18°C to 23°C. The weight, nitrogen concentration, and GE concentration of urine were determined every 2 d. Urinary nitrogen concentrations were determined using an automatic Kjeldahl analyzer (method 990.03) as described in AOAC [12] and urinary GE concentrations were determined using the procedure described by Kim et al [13].

Experimental data were analyzed using the MIXED procedure (SAS Inst. Inc., Cary, NC, USA). The statistical model included day, supplementation of 6 *N* HCl, and interaction between day and supplementation of 6 *N* HCl as fixed variables, and the day was the repeated term in this model. The values of least squares mean were calculated. Orthogonal polynomial contrasts were used to test the linear and quadratic effects of day and the interaction between day and supplementation of 6 *N* HCl. Each plastic container was an experimental unit. Statistical significance and tendency were declared at alpha less than 0.05 and 0.10, respectively.

### Exp. 2. A maximum pH for nitrogen preservation in pig urine

Urine samples were collected from 10 barrows ([Landrace× Yorkshire]×Duroc) with a mean BW of 41.2±2.1 kg with no acid in the urine collection containers and were filtered using cotton cloth (0.5 mm pore size) to remove impurities. The samples were stored in a sealed container at −20°C. Three urine samples were selected to contain variable nitrogen concentrations of 0.12, 0.53, and 0.94 g/100 mL. To determine of the maximum pH for nitrogen preservation, six 100-mL aliquots from each urine sample were added with 6 *N* HCl to achieve pH values of 0.6, 1.1, 2.2, 4.7, 7.1, and 9.3 in 18 plastic containers. The plastic containers with the urine of various pH values were placed on a laboratory table at room temperature for 5 d. Nitrogen concentrations were analyzed (method 990.03; AOAC, 2019) at the beginning and after 5 d to determine the nitrogen losses from the pig urine.

A break point of a pH value for nitrogen preservation was estimated by a one-slope broken-line model using the NLIN procedure of SAS (SAS Inst. Inc., USA). A plastic container was an experimental unit and statistical significance was declared at an alpha less than 0.05.

### Exp. 3. A minimum amount of HCl required for nitrogen preservation in pig urine

Five urine samples were selected from 10 samples of Exp. 2 to obtain variable nitrogen concentrations of 0.12, 0.26, 0.53, 0.61, and 0.94 g/100 mL. The pH changes of each 100 mL of urine samples were measured every addition of 0.2 mL of 6 *N* HCl using a pH meter (SevenEasy pH Meter S20; Mettler Toledo, Columbus, OH, USA).

The NLIN procedure (SAS Inst. Inc., USA) was employed to develop exponential equations for estimating urine pH by the volume of added HCl in each urine sample with various nitrogen concentrations. An equation for determining the minimum amount of 6 *N* HCl for nitrogen preservation in urine was generated by the REG procedure of SAS (SAS Institute, 2012) with 6 *N* HCl concentrations in urine as a dependent variable and nitrogen concentrations in urine as an independent variable. A plastic container was an experimental unit and statistical significance was declared at alpha less than 0.05. Additionally, the amounts of 6 *N* HCl required for nitrogen preservation were calculated based on actual daily nitrogen excretion data from 9 published experiments and 8 unpublished experiments conducted in our laboratory.

## RESULTS AND DISCUSSION

### Exp. 1. Nitrogen and energy contents in pig urine under acidic condition

The amount of nitrogen in the urine showed a linear interaction (p<0.001) between acid supplementation and time (Table 1). The amount of nitrogen in the urine supplemented with distilled water decreased linearly with time, whereas that supplemented with 6 *N* HCl remained constant regardless of the time. The amount of GE in the urine had a tendency for linear interaction (p = 0.053) between acid supplementation and time. The amount of GE in the urine supplemented with distilled water tended to decrease linearly with time, whereas that supplemented with 6 *N* HCl remained constant regardless of the time. These results indicate that urea was hydrolyzed to ammonia molecules and volatilized under alkaline conditions, but not under acidic conditions. In the energy metabolism experiments, urine is collected daily basis with acids in the collection containers and subsamples are stored in the freezer [3,4,9,14]. The present results also indicate that urine samples can be stored at room temperature for at least 10 d without nitrogen or energy volatilization if the urine pH is kept below 2. Under acidic conditions, a large quantity of hydrogen ions traps ammonia as ammonium ions [15,16] that do not dissociate into hydrogen ions and ammonia [17].

As urea is a major energy source in urine [18], the constant urine energy under acidic conditions regardless of the time is reasonable. The amounts of energy at d 0 were expected to be the same between the 2 groups. However, the amount of energy in the distilled water-added urine was 30.6% less (12.2 vs 17.6 kcal; Table 1) than that in the acid-added urine on d 0. This unexpected result is most likely due to the volatilization of ammonia from distilled water-added urine during the lyophilization procedure before GE determination. Thus, the addition of acids to pig urine is critical to prevent ammonia volatilization from urine during collection and sample drying processes for an accurate determination of urinary GE.

### Exp. 2. A maximum pH for nitrogen preservation in pig urine

**Figure 1:**
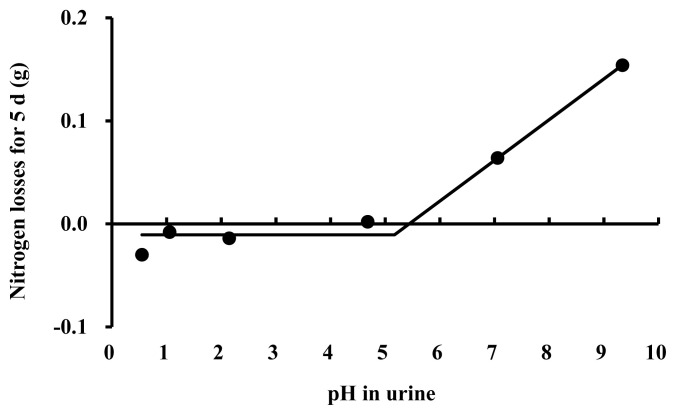
A broken-line analysis of nitrogen losses for 5 d at 6 urine pH values acquired by different inclusion rates of 6 *N* hydrochloric acid into the 100 mL of each urine sample (Exp. 2). Each data point represents the least squares mean of 3 observations. The amount of nitrogen was 0.53 g on average in 100 mL urine at the beginning. A one-slope broken-line model of nitrogen losses for 5 d indicates that a maximum pH of 5.12 is needed to prevent nitrogen volatilization from the pig urine. The break point was estimated based on following equation: Y = 0.04×(X–5.12)–0.01 where X is more than 5.12 (standard error = 0.712 and p<0.01) in Exp. 2.

Based on the one-slope broken-line analysis (Figure 1), nitrogen in pig urine was not volatilized at pH below 5.12 (R^2^ = 0.98, standard error = 0.71, and p<0.01). The present results demonstrate that the use of acid in the urine collection container to keep pH below 5 would be sufficient for nitrogen preservation. This result agrees with the previous suggestions that the pH of the urine should be kept below 5 to avoid nitrogen volatilization [19,20]. Although urine pH values are rarely measured in energy metabolism or nitrogen balance experiments, the pH of collected urine may have exceeded 5 due to insufficient addition of acid to the urine containing a large quantity of urea. Thus, the addition of a sufficient amount of acid to make the pH less than 5.12 is critical for nitrogen preservation in pig urine.

### Exp. 3. A minimum amount of HCl required for nitrogen preservation in pig urine

**Figure 2:**
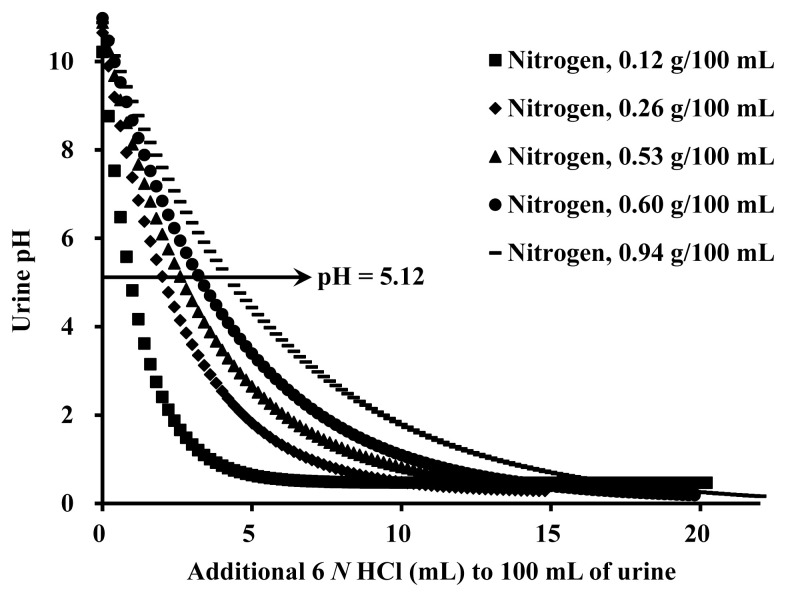
Urine pH changes by the addition of 6 *N* hydrochloric acid (HCl) to urine (Exp. 3). Exponential models were developed for each urinary nitrogen concentration: Y = −9.28+9.75×(1+e^−0.81^^×^^X^), with p<0.001 for nitrogen 0.12 g/100 mL; Y = −10.16+10.41×(1+e^−0.38^^×^^X^), with p<0.001 for nitrogen 0.26 g/100 mL; Y = −10.26+10.57×(1+e^−0.30^^×^^X^), with p<0.001 for nitrogen 0.53 g/100 mL; Y = −10.80+10.89×(1+e^−0.24^^×^^X^), with p<0.001 for nitrogen 0.60 g/100 mL; Y = −10.98+10.93×(1+e^−0.18^^×^^X^), with p<0.001 for nitrogen 0.94 g/100 mL. The required concentrations of 6 *N* HCl to achieve a urine pH less than 5.12 were 0.92, 2.01, 2.61, 3.24, and 4.20 mL/100 mL for urinary nitrogen concentrations of 0.12, 0.26, 0.53, 0.60, and 0.94 g/100 mL, respectively.

**Figure 3:**
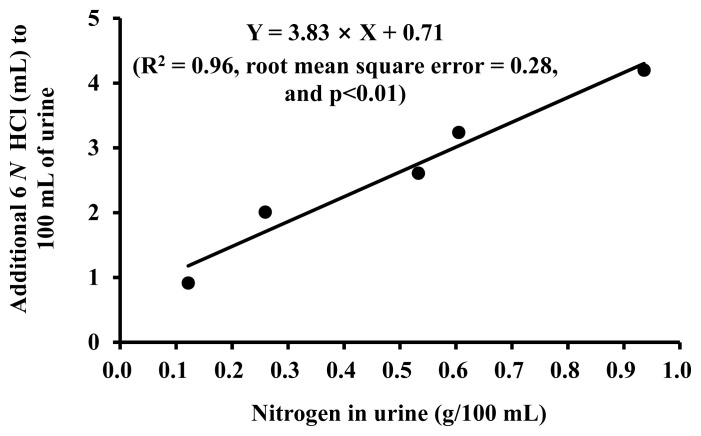
Minimum amounts of 6 *N* hydrochloric acid (HCl) required for nitrogen preservation for 100 mL pig urine based on urine nitrogen concentration (g/100 mL; Exp. 3). The plotted data were based on the x-axis values for pH = 5.12 and urinary nitrogen concentrations presented in Figure 2.

Exponential models were developed for each of the 5 urine samples to estimate urine pH changes by adding 6 *N* HCl (Figure 2). Using these models, the amount of 6 *N* HCl required for 100 mL of urine to achieve the pH 5.12 was calculated for each of the 5 urine samples with various nitrogen concentrations. Based on these data, an equation was generated using nitrogen concentration in urine (g/100 mL) as an independent variable to determine a minimum amount of 6 *N* HCl required for nitrogen preservation in pig urine (Figure 3).

Nitrogen concentrations in pig urine are affected by water intake and dietary fiber concentrations due to changes of water absorption to the circulation system of pigs and urinary water excretion [2,7,21]. However, these 2 factors do not affect absolute amounts of urinary nitrogen excretion. For the calculation of an amount of HCl required for nitrogen preservation in urine, the amount of excreted urinary nitrogen should be considered. High dietary protein contents [22–24] and an imbalance of dietary amino acids [25,26] cause an elevation of urinary nitrogen excretion in pigs.

Based on actual data for daily nitrogen excretion, the amount of 6 *N* HCl required for nitrogen preservation was calculated to be 48 to 240 mL per day for the largest daily urinary nitrogen excretion for each BW range (Table 2). In this calculation, the maximum quantity of daily nitrogen excretion (g/d) for each BW range was multiplied by the required amount of 6 *N* HCl for nitrogen preservation per gram of urinary nitrogen (3.83 mL/g) which is the slope in Figure 3. In energy metabolism or nitrogen balance experiments, the amount of urinary nitrogen excretion is largely variable and difficult to predict accurately. Therefore, a sufficient amount of acids should be used for nitrogen preservation based on the calculations provided in this work.

## CONCLUSION

The amount of nitrogen and gross energy in pig urine remains constant at room temperature if the urine is highly acidic. The urine pH needs to be below 5.12 to inhibit nitrogen volatilization from pig urine. Although urinary nitrogen excretion is largely variable depending on many factors including experimental diets and growth stage of pigs, and the amount of 6 *N* HCl required for nitrogen preservation may be up to 240 mL per day for a 110-kg pig depending on urinary nitrogen excretion.

## Figures and Tables

**Table 1:** Effects of hydrochloric acid supplementation to pig urine on nitrogen and gross energy (GE) contents at room temperature for 10 days, Exp. 1^1),2)^

Item	Hydrochloric acid (d)						Distilled water (d)						SEM	p-value^3)^				
		
	0	2	4	6	8	10	0	2	4	6	8	10		HCl	L	Q	HCl×L	HCl×Q
Urine weight (g)	248	238	231	223	214	199	251	237	228	222	211	203	2	0.868	<0.001	0.970	0.927	0.014
Nitrogen concentration (%)	0.574	0.602	0.626	0.643	0.668	0.707	0.563	0.478	0.442	0.403	0.366	0.326	0.071	<0.001	0.002	0.198	<0.001	0.451
Nitrogen amount (g)	1.43	1.44	1.45	1.43	1.43	1.40	1.41	1.13	1.01	0.90	0.77	0.66	0.16	<0.001	<0.001	0.234	<0.001	0.051
Corrected nitrogen^4)^ (%)	0.574	0.571	0.575	0.574	0.572	0.571	0.563	0.452	0.404	0.359	0.311	0.265	0.064	<0.001	<0.001	0.124	<0.001	0.130
GE concentration (kcal/kg)	70.8	77.2	74.1	77.7	83.7	92.4	48.3	45.7	42.6	44.2	46.5	48.9	7.3	<0.001	<0.001	0.197	0.001	0.800
GE amount (kcal)	17.6	18.4	17.1	17.3	18.0	18.4	12.2	10.8	9.7	9.8	9.8	9.9	1.6	<0.001	0.199	0.399	0.053	0.363
Corrected GE^5)^ (kcal/kg)	70.8	73.2	68.1	69.3	71.7	74.6	48.3	43.2	39.0	39.4	39.6	39.7	6.6	<0.001	0.259	0.276	0.042	0.564

1) Each least squares mean represents 5 observations.

2) The room temperature for 10 d ranged from 18.3°C to 25.9°C.

3) HCl, supplementation of 6 *N* HCl in urine; L, linear effects of day; Q, quadratic effects of day; HCl×L, the interaction between supplementation of 6 *N* HCl in urine and linear effects of day; HCl×Q, the interaction between supplementation of 6 *N* HCl in urine and quadratic effects of day.

4) Corrected nitrogen concentration in urine at d 2, 4, 6, 8, and 10 (%) = weight of urine at a specific day (g)×nitrogen concentration in urine at a specific day (%)÷weight of urine at d 0 (g).

5) Corrected GE concentration in urine at d 2, 4, 6, 8, and 10 (%) = weight of urine at a specific day (kg)×GE concentration in urine at a specific day (kcal/kg)÷weight of urine at d 0 (kg).

**Table 2:** The ranges of daily urinary nitrogen excretion (g/d) according to body weight (BW) and required amounts of 6 *N* hydrochloric acid (HCl) for urinary nitrogen preservation, Exp. 3^1)^

BW (kg)	Number of observations	Maximum nitrogen (g/d)	Minimum nitrogen (g/d)	Mean (g/d)	Required 6 *N* HCl^2)^ (mL/d)
0 to 20	88	12.6	2.1	4.6	48
20 to 40	101	29.6	2.8	15.1	113
40 to 60	270	49.6	5.3	20.0	190
60 to 80	225	59.1	7.0	23.2	227
80 to 110	121	62.7	7.1	27.9	240

1) Data were from 9 published experiments and 8 unpublished experiments that measured daily urinary nitrogen excretion.

2) The amount of 6 *N* HCl required for urinary nitrogen preservation was calculated by multiplying the maximum quantity of daily nitrogen excretion for each BW range with the required amount of 6 *N* HCl for nitrogen preservation per gram of urinary nitrogen (3.83 mL/g) which is the slope in Figure 3.
